# The Colony for Cripples, Chailey

**Published:** 1915-04-03

**Authors:** A. F. London

**Affiliations:** London House, 32 St. James's Square, S.W.


### THE EDITOR'S LETTER-BOX.

### The Colony for Cripples, Chailey.

*To the Editor of* The Hospital.

Sir,—As President of the Heritage Colony for Cripples at Chailey, Sussex, I venture to hope that the public will generously support the military wards for the reception of wounded soldiers at the Boys' Heritage which have been recently established, and named after our chief patroness, H.R.H. Princess Louise, Duchess of Argyll. The care, employment, and training of wounded sailors and soldiers is one of the pressing needs of the moment. The Heritage Boys' School is particularly suitable for the reception and accommodation of wounded men. Those now with us, and those who have already left, are ample proof of this statement.

On Sunday last, making the tour of these military wards, when paying a surprise visit to these splendid fellows who have been wounded in the service of their King and country, and, at the same time, making a farewell visit to the cripples before going to the Front, I was immensely struck with the suitability of the buildings, the surroundings, and the whole environment for the treatment of wounded men. The fine library and rooms opening off it have been transformed into medical wards, of which any of our great hospitals might well be proud; with bathroom, dispensary, washing arrangements, dining-room, smoking-room, kitchens and other offices, all on the ground floor; the whole opening on to the sunny quadrangle, facing south-west, while all round the school stretches the glorious open common. Doubtless the healthy position of these schools, added to the skill and care of the cripples shown by the entire staff, explains the small percentage of sick leave among the children from their daily lessons. In order to show what the schools themselves have accomplished at this crisis I may mention that two of the cripple boys are now serving with the Colours—one in the King's Royal Rifles, and the other in Kitchener's Army.

Everyone now recognises the advisability of surrounding cripple children with the utmost medical skill and nursing care, as well as giving them the fullest educational advantages. I know of no place in the kingdom where the results are equal to those attained in our schools at Chailey. For our wounded soldiers there could be no healthier and happier or more wholesome surroundings. To see them gardening with the boys, working with them in the craft shops, joining in the services in chapel, is indeed an inspiration, and it is my firm belief that this scheme of educative convalescence for our brave wounded men will ripen and develop into a great national scheme.

Our beautiful little chapel still needs several additions, and we will gladly supply a list to those who would like to make gifts as memorials or thankofferings in connection with this terrible war. There must be many who feel that such gifts would be most suitably placed, and I can guarantee, most reverently guarded, in such surroundings as these. I can further guarantee that those who support these military wards for wounded soldiers may rest assured that their money will be well spent, under the supervision of those who have had long experience in dealing with the disabled. A considerable sum might be raised by offertories from the chapels of our great public and private schools, and I should much like to see this idea developed this Easter. We have all great tasks ahead of us, but to such work as this let us each give all that we can—schoolboys, schoolgirls, men and women, of strength, of time, of work, of money. May lavish sympathy and many hundreds of pounds find their way this Eastertide to the Princess Louise military wards at Chailey, Sussex, is my wish as President of the Heritage Colony.—Yours very truly,

A. F. London.

London House, 32 St. James's Square, S.W.

P.S.—Gifts specially needed at the moment are bagatelle boards, piano, gramophone and records, clothing of all descriptions, books and magazines, revolving shelter for use of men in the garden, loan of motors for those of the wounded men who are unable to walk. All communications should be addressed to Mrs. C. W. Kimmins, The Old Heritage, Chailey, Sussex.

